# Understanding Threats to Young Children’s Green Space Access in Unlicensed Daycare Centers in Japan

**DOI:** 10.3390/ijerph17061948

**Published:** 2020-03-16

**Authors:** Christoph D. D. Rupprecht, Lihua Cui

**Affiliations:** 1 FEAST Project, Research Institute for Humanity and Nature, Kyoto, 603-8047, Japan; 2 Graduate School of Agriculture, Kyoto University, Kyoto, 606-8502, Japan; lihua.cui.36x@st.kyoto-u.ac.jp

**Keywords:** greenspace, outdoor play, development, health, environmental justice, East Asia, urban planning, nursery school, kindergarten, 認可外保育施設

## Abstract

Access to green space (GS) is vital for children’s health and development, including during daycare. In Japan, deregulation to alleviate daycare shortages has created a new category of so-called unlicensed daycare centers (UDCs) that often lack dedicated GS. UDCs rely on surrounding GS, including parks, temples and university grounds, but reports of conflicts highlight the precarity of children’s well-being in a rapidly aging country. Knowledge about GS access in Japanese UDCs remains scarce. Our mail-back survey (n = 173) of UDCs and online survey (n = 3645) of parents investigated threats to GS access during daycare across 14 Japanese cities. Results suggest that UDCs use a variety of GS and aim to provide daily access. Caregivers are vital in mediating children’s access, but locally available GS diversity, quality and quantity as well as institutional support were perceived as lacking. Parents did not rank GS high among their priorities when selecting daycare providers, and showed limited awareness of conflicts during GS visits. Implications of this study include the need for caregivers and parents to communicate and collaborate to improve GS access, and the importance of strong public investment into holistically improving GS diversity, quality and quantity from the perspective of public health and urban planning.

## 1. Introduction

The old idiom “children should be seen, not heard”, is at odds with contemporary views on children’s developmental needs. Yet this attitude still seems to be reflected in the way residents in aging Japan oppose the construction of new daycare centers in urban areas, even though the rise of women’s participation in employment is driving a need for more daycare. Allowing daycare centers to be integrated into office buildings is one way the Japanese government has tried to tackle this problem [1]. Yet what consequences this might have for children’s nature contact, vital for their development, is unclear. In a country already struggling with low urban green space (GS) provision amidst rising park maintenance costs and sinking tax income, a better understanding of children’s access to GS during daycare is urgently needed to address this issue of environmental and intergenerational justice. In this paper, we report on practices and attitudes of the two main stakeholder groups, daycare caregivers and parents, responsible for securing nature contact for children during daycare. We argue that current public GS provision is inadequate to meet children’s needs, emphasize the importance of caregivers in negotiating access to GS, and reflect on the role of parents and their attitudes as background drivers. To conclude, we provide some suggestions for concrete steps to improve children’s access to GS in Japanese unlicensed daycare centers (UDCs).

In recent years, a strong consensus has emerged across children’s health research that sees nature contact and outdoor play as vital for healthy mental and physical development [2,3,4,5,6,7]. Opportunities for unstructured play, learning about and making sense of the natural world through encounters with animals and plants, social interactions with peers as well as time and space for reflection have all been linked with sound social, mental, emotional, and physical health and development. With large, well-designed studies the evidence base for the importance of such opportunities today is stronger than ever before. Yet researchers have also shown that the time children are allowed to, or choose to, spend outdoors and specifically in contact with nature is declining at an alarming rate across industrialized countries [8,9,10,11,12]. This has raised concerns that children’s development, especially in urban areas, is at risk through what has been termed nature-deficit disorder [13,14]. While some studies of children’s physical abilities provide evidence for a decline, researchers have also suggested that the roots of the problem may lie deeper [15].

In this study we apply a simplified conceptual model of drivers influencing children’s access to GS in daycare with three main components: parents, caregivers, and local context. In general, parents are seen as mediators of their children’s time and activities. Given the importance of nature contact for children’s health, one could assume parents are strong advocates of better access. However, the affordances children are given for unstructured, unsupervised outdoor play seem to be declining considerably in comparison to prior generations. For example, research on play in vacant lots suggests that this practice was common in the past, but due to safety concerns parenting has become considerably more restrictive [16]. Indeed, the trend of constant adult supervision and a low-risk parenting paradigm may deny children’s need for experiencing adventure and risk [17], suggesting that ‘the role of “parent” constitutes a barrier to children’s access to challenging places and experiences’ [18]. Rather than being simple proponents of better access to nature for children, parents can thus mediate access in ways that enables or limits it. Parents can also influence children’s access indirectly, be it through the values they convey or the choices they make. In particular, the relationship between parents and caregivers in daycare has been shown to be complex [19]. When considering what drives children’s access to GS during daycare, it is therefore necessary to understand parental attitudes towards nature contact. 

In comparison to parents, caregivers are only responsible for children’s nature contact during the limited hours of daycare contact. However, daycare hours often reflect parents’ work hours and fall into the prime outdoor play hours of the day. In addition, researchers have argued that parents increasingly shift educational responsibilities onto institutions and paid providers, resulting in a care deficit [20,21,22,23]. Daycare is thus an important space to consider when examining children’s nature contact. Within this space, daycare centers plan activities around recommended guidelines, but also enjoy some discretion. Particularly the choice of off-premise GS, what activities to undertake, and what affordances to give children is likely to affect when, where and how children interact with nature. Physical factors such as the GS available in the vicinity of the daycare center can be seen as a necessary but not sufficient aspect in securing GS access. Social factors such as interaction with other GS users are similarly mediated through caregivers. Caregiver practices and attitudes are thus central to understanding children’s access to GS during daycare.

**Figure 1:**
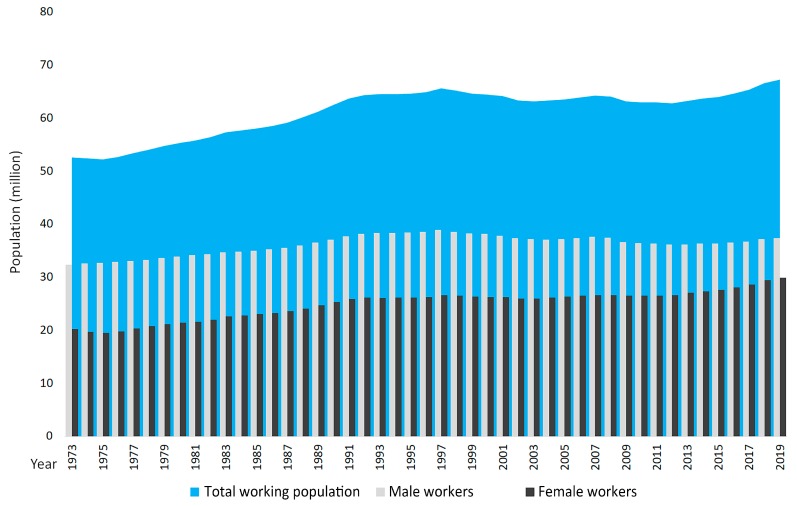
Rise in women’s share of the total working population 1973–2019 [26].

Local context, the third component in our conceptual model, provides the background for how parents and caregivers mediate children’s GS access. The rapid demographic transition Japan is experiencing (Figure 1) has implications for the historically ambivalent view of daycare in Japanese society [24]. As its society is aging, structural issues such as a shrinking tax base and thus shrinking financial means for maintenance and creation of GS are emerging as challenging issues, even though climate adaptation and residents’ preferences are increasing demand for GS [25]. Meanwhile, densely built urban areas and resistance of residents due to noise concerns are making the establishment of new daycare centers with outdoor play facilities difficult. However, the demographic transition is also behind the governments’ strategy to increase women’s participation in the workforce, which in turn is one factor in a larger shift of women’s emancipation in society that contributes to increased demand for daycare facilities (Figure 1).

The lack of daycare has been an ongoing topic, leading at times to harsh criticism of the Japanese government. In 2016, a blog post by a woman being denied daycare for her child made national news, in which she expressed her frustration by writing “Die, Japan” [27,28]. This brought into sharp light the irony of the government’s ongoing yet unsuccessful efforts to raise the birthrate (currently far below the replacement rate at 1.42), repeatedly encouraging young Japanese to have more children, despite a background of increased irregular employment and lack of daycare to accommodate families with two working parents [29,30,31]. As noted above, one of the difficulties the government faced in expanding daycare is the often-vocal resistance against perceived noise issues. This phenomenon marks a shift in society from a silent mutual understanding about children making noise being a matter of fact, to a peculiar form of not-in-my-backyard thinking, all amidst media discussions of the possibility of the Japanese becoming ‘extinct’ [32]. In response, the government deregulated the daycare center sector by introducing a new type (Table 1), so-called unlicensed daycare centers (UDCs, also called non- or un-authorized daycare facilities, in Japanese *ninkagaihoikushisetu*/認可外保育施設) [1,33,34]. 

In contrast to the three major, licensed types of daycare centers in Japan (Table 1), UDCs are not required to provide dedicated outdoor space. This allows UDCs to be established in existing residential or commercial office buildings, under the assumption that children will be given access to GS through visits to parks and other public or private spaces (including university grounds, temples, forests etc.). Such use of GS in the vicinity is not new and also practiced by daycare centers with dedicated outdoor spaces. However, it is unclear to what degree the resulting increase in GS demand was anticipated, particularly in central urban areas convenient for working parents but often with less GS than residential areas. In 2016, Hokkaido University, known and appreciated by residents for its extensive park-like campus, made headlines for banning children from its lawns, a ban enacted following a sharp increase in maintenance costs due to increased use [35]. This incident raised several questions: how are daycare centers and children in the area able to access GS? What is the role and responsibility of public GS owners? How frequent are such conflicts across Japan? How are caregivers navigating such conflicts, and what support are they receiving? Are parents aware of such conflicts? Overall, the literature on these issues is very sparse. Some research published in Japanese has examined outdoor play and public park use by Japanese daycare centers [36,37,38,39,40,41,42,43,44,45,46]. Two studies focusing on Yokohama (Yokohama day nurseries or *hoikushitsu*) and on from Sapporo included UDCs and highlighted their dependence on surrounding parks [40,42,45].

Otherwise noteworthy are two studies by Miyaji and colleagues who found in a large-scale survey of local governments that only 18% provide child-care support services utilizing parks, but generally high satisfaction in a survey of users of such services [44,47]. In this paper we endeavor to answer some of the above questions and thus contribute to a better understanding of UDCs and GS use in Japan. Addressing these questions will help to inform urban planning and public health policies in Japan, particularly GS planning and GS provision policies for children. Moreover, given that countries such as South Korea and China are on similar demographic trajectories, findings may prove useful in these contexts as well. The study thus contributes to the site-specific, national and international discourse. This paper examines the following research questions:

(1) What is the role of UDCs in providing children with access to green space?
  (a) What are the practices of UDCs in providing green space access?
  (b) What is UDC caregivers’ perception of the role of green space for children’s development?
  (c) What is the UDCs willingness to pay for access to green space?
  (d) What factors influence UDCs provision of green space access?
(2) What is the role of parents for children’s access to green space during daycare?
  (a) What factors influence parents’ choice of daycare?
  (b) What is parents’ perception of children’s access to green space?
  (c) What is parents’ willingness to pay for access to green space?
  (d) What factors influence parents’ attitude towards green space access?
(3) What are the implications for policy and stakeholders?

We find that UDCs use a variety of GS and aim to provide daily access. Caregivers are vital in mediating children’s access, but locally available GS diversity, quality and quantity as well as institutional support were perceived as lacking. Parents play a lesser role, as they did not rank GS high among their priorities when selecting daycare providers, and showed limited awareness of conflicts during GS visits. We finish by discussing the implications of this study, including the need for caregivers and parents to communicate and collaborate to improve GS access, and the importance of strong public investment into holistically improving GS diversity, quality and quantity from the perspective of public health and urban planning. 

## 2. Materials and Methods 

### 2.1. Study Sites

**Figure 2:**
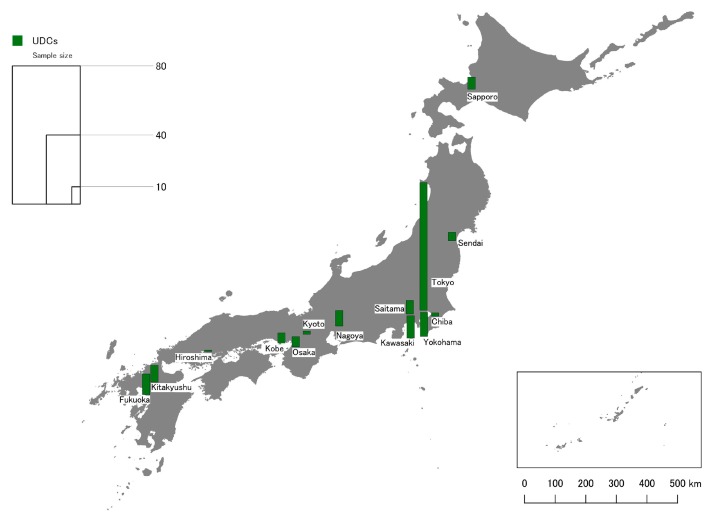
Study site locations and data samples for UDC survey.

**Figure 3:**
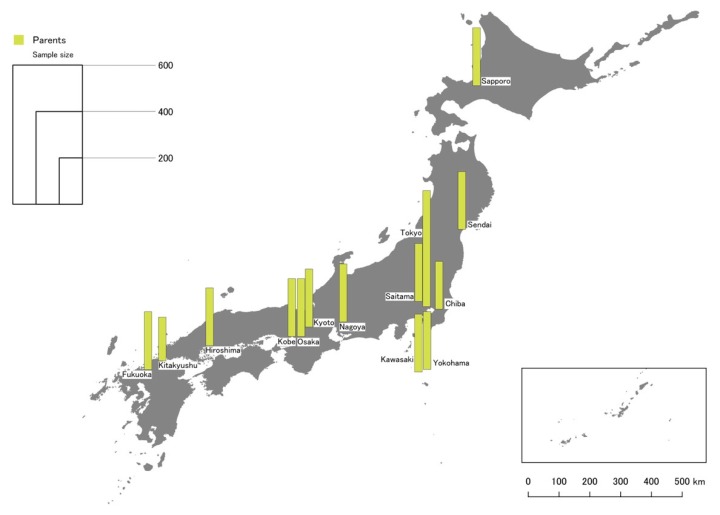
Study site location and samples for the parent survey.

This paper focused on 14 major Japanese cities (Table 2), which account for a majority of all UDCs in Japan as well as areas with the highest demand for daycare. They were selected to provide a representative overview across a range of contexts, including differences in city age, population size, population density, GS per capita and geographic location (Figure 2 and Figure 3). In total the cities are home to about 30 million Japanese urban residents.

### 2.2. Data Collection, Survey Instruments and Data Analysis

#### 2.2.1. Survey of Unlicensed Daycare Centers (UDCs)

In contrast to other daycare center types, UDCs have some characteristics that make them comparatively difficult to survey. Regulation and oversight of UDCs happens in principle at the municipal level, and to our knowledge there is no publicly available up-to-date central database or register. For this reason, a database of 1,840 UDCs including available data such as name, address, capacity etc. was compiled from publicly available lists on the 14 municipal governments’ websites from 2017 to 2018. After data cleaning, 1,820 UDCs were identified as survey targets. In the case of Tokyo, Tokyo Metropolitan Area Certified Daycare Centers (東京都認証保育所) were excluded, as they more closely resemble traditional daycare types and are not listed as UDCs on the government website. With the assistance of ResearchWorks, a professional Japanese survey provider, a mail-back survey of these UDCs was conducted in 2018 across all 14 cities. Measures to increase the response rate included sending the survey documents in a transparent envelope with the cover sheet legible from the outside, as well as follow-up reminder postcards. Follow-up phone calls were not conducted due to budget restrictions. The survey was approved by the home institution’s research ethics committee (RIHN2017-3).

The survey instrument (see Supplementary File S1) consisted of a combined cover and informed consent sheet as well as a two-page double-sided question sheet with 22 questions in four parts: (1) basic data on the UDC including year of establishment, capacity, staff numbers, licensing information and presence of dedicated GS inside the facility; (2) the UDCs’ basic practices regarding GS access outside of the facility, including frequency of visits, types of GS used, non-GS used, and length of visits; (3) context of UDCs’ GS practices including plans for GS access, potential for improvements, contacts with GS managers, problems encountered, support received from the municipality, and willingness to pay for access; (4) caregiver’s attitudes towards the importance of GS access for 13 aspects of children’s health and development, as well as a self-evaluation of the actual GS used based on the same 13 aspects. Question types included multiple choice, Likert-scale, and open comment qualitative questions. Native Japanese speakers helped to ensure the survey instrument was linguistically correct and easy to read. Questions drew on previous research about children’s use of parks during daycare, in particular the 13 aspects of children’s health and development, were modified and extended based on work by Miwa and colleagues [40].

#### 2.2.2. Survey of Parents with Children in Daycare

The survey was conducted across the 14 study sites through an online survey coordinated by Rakuten Insight, a major Japanese online polling service. Respondents were recruited from the service’s panel, consented to and received compensation for their participation in accordance to the service’s policies (the amount of compensation is not disclosed by the company, but is less than ¥1,000). The service does not provide classic response rate data, but rather surveys the sample until the target sample size is reached or the panel is exhausted. Responses were collected over a period of two weeks in 2019 in a two-step process. First, a screening survey identified respondents who live in the study sites with a child aged 5 years or under currently using daycare center services. These respondents were then asked to complete the full survey instrument. Respondents were able to complete the survey in on a device of their choice (personal computer, tablet, smart phone). The survey was approved by the home institution’s research ethics committee (RIHN2017-3).

The main survey instrument (see File S2) consisted of five parts: (1) respondents’ socio-demographic data (housing type, educational attainment, household income, post code); (2) information about their daycare center of choice, including reasons for choosing this daycare center, distance from home, work and frequently used public transport station, presence of dedicated outdoor play space inside the facility; (3) GS access during daycare, including access frequency, knowledge about problems encountered during access, and willingness to pay for access; (4) respondents’ (parents’) attitudes towards the importance of GS access for 13 aspects of children’s health and development, as well as their perception of the quality of the actual GS used during daycare based on the same 13 aspects; (5) children’s use of GS outside of daycare as well as respondents’ perception of the quality of the GS used based on the 13 aspects above. Question types included multiple choice, Likert-scale, and short-form open comment qualitative questions. Native Japanese speakers helped to ensure the survey instrument was linguistically correct and easy to read. Questions were designed to mirror those used in the daycare center survey, while providing additional context in the form of motivations for choosing daycare as well as limited information about children’s access to GS outside of daycare.

#### 2.2.3. Data Analysis

Data was analyzed using descriptive and inferential statistics following procedures described by Field and colleagues [52], primarily using jamovi [53]. The map of study locations and sample size (Figure 1 and Figure 2) was created with QGIS [54], but no spatial analysis was performed for this study. For some analysis, non-parametric tests were used as analysis indicated that the data did not fulfill the assumptions of parametric tests. In principle, only statistically significant results (*p* < 0.05) with non-trivial effect sizes are reported, and effect size is provided in text to focus on noteworthy results. The reliability of core scales (importance of GS for children’s health and development) was high (Cronbach’s alpha > 0.9). The analyzed data set is available as supplementary material (File S3, File S4).

## 3. Results

### 3.1. Unlicensed Daycare Centers’ Perspective on Children’s Access to Greenspace

#### 3.1.1. Sample Characteristics

A sample with responses of 173 UDCs were collected (response rate: 9.5%). UDCs showed a wide range of characteristics, from their date of establishment to staff number, number of children supervised and opening hours (Table 3). A majority had no dedicated GS on the premises.

#### 3.1.2. UDC’s Practices in Providing GS Access

Most respondent UDCs reported using off-premise GS almost daily, or at least two to three times per week, while only very few never or rarely did so (Table 4). Parks in the neighborhood were most frequently used, followed by temples/shrines, other public or private GS, and riverside spaces. UDCs showed a wide range in both number and diversity of GS used. Most visits lasted between 30 min to one hour. Non-GS (roads, local shopping arcades etc.) were only infrequently used.

UDCs reported taking the issue of GS access into account when establishing the facility, including selection of the daycare center site and proximity to parks. Responses showed that many UDCs in principle aim for visiting GS every day, with the exception of rain, strong heat or otherwise unsuitable weather. While most (62%) UDCs did not report changes in outdoor activities, some reported a range of changes being made to facilitate GS access (change of location or visit time, response to parent requests etc.). Only a minority of UDCs (18%) had previously communicated with the manager or owner of the GS regarding its use for the daycare’s children. Topics of conversation in these cases included establishing contact to inform the manager/owner about planned use, enquiries around specific GS uses (play gear, water features etc.), participation in regular collective cleaning activities, and concerns about safety and litter. Almost all UDCs reported receiving no support from their respective municipal governments in providing GS access. In the few cases where support was provided (7%), it consisted mainly of providing park information (play gear, natural features such as seasonal flowers, neighborhood park map) or permission to use park space for special event activities. Only one UDC reported being invited to a workshop where they were able to voice their preferences and ideas as park stakeholders to be reflected in a park overhaul. 

A majority (70%) of UDCs responded they had not experienced any conflict with GS managers, other users, or neighbors that might impede children’s access. In contrast, the issues reported by UDCs which had encountered conflicts (23%), aside from a variety of others, broadly fell into three categories: noise complaints, conflicts with other users, and safety concerns. Complaints about the volume of the children’s voices (both at the GS and on the way there) were the most prominent among reported conflicts, in particular those raised by elderly neighbors. Conflicts with other users included space constraints and crowding (sometimes due to multiple daycare centers visiting a park simultaneously), other park users being angry about and questioning the use of GS by UDCs, and hostile attitude towards children and caregivers. For example, UDCs reported repeatedly becoming targets of elderly men complaining about a perceived lack of greeting or manners. Some of these conflicts also overlapped with safety concerns raised. Besides conflicting play by different age groups and age-inappropriate play gear, litter and specifically cigarette butts were reported, in some cases alongside hostile attitude by smokers or smokers smoking outside of designated zones. In their responses, UDCs noted they and their caregivers are careful to devote time and effort to maintaining good community relations and avoiding conflicts. For example, multiple UDCs reported participating in community cleaning activities, informing managers about their intended GS use, and actively greeting and communicating with other park users. 

Some of these concerns were also reflected in the responses of UDCs to the question how children’s outdoor play might be improved. Providing an ideal environment for children’s physical and mental health stood at the foreground, but some tensions were present in how to best achieve this. Many improvements aimed at making children’s play safer. Ideas for improvements during time spent inside GS included among others taking care of litter including through heightened awareness and cooperation by community members, providing safe spaces for activities by children aged zero to two years such as crawling on lawn, eliminating sources of injuries including dangerous insects and weeds, and lower children per caregiver ratio. Better embedding GS in their urban fabric was also suggested, including reducing traffic accident risk by avoiding park placement next to high traffic roads, designs to avoid children dashing out into traffic, and creating save travel routes to and from GS. In contrast, other UDCs emphasized needs beyond safety, as well as the potential dangers of focusing only on safety concerns such as a plethora of rules and restrictions that risk impeding child development by lowering the quality of play and experiences. Needs of children to express themselves in their diversity were noted together with a desire to provide a range of diverse nature experiences. Responses thus emphasized both the need for more GS quantity and more GS diversity (in particularly within parks), with rich natural features being prioritized over play gear. 

#### 3.1.3. Caregivers’ Perception of the Role of Greenspace for Children & Capacity of Existing Greenspace to Fulfill this Role

**Figure 4:**
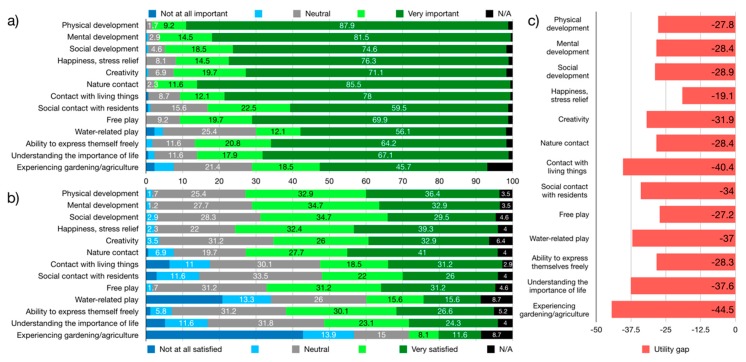
Caregivers perception of GS: (**a**) importance of outdoor play for children aged five or under in regard to…, (**b**) satisfaction with regularly used greenspace in regard to…, (**c**) utility gap between satisfaction and importance.

A large majority of UDC caregivers (>80%) placed a high importance on the role of outdoor play for children’s development, with physical development, nature contact, and mental development being ranked as most important benefits (Figure 4). In contrast, only slightly more than a third of caregivers was very satisfied with how well GS they regularly used were able to fulfil this role. The utility gap, which visualizes the discrepancies between caregivers’ expectations and reality, was largest for contact with living things and experiencing gardening/agriculture, and exceeded 20 points for all items except happiness/stress relief. Regularly used GS failed to fulfil or exceed caregivers’ expectations.

#### 3.1.4. UDCs Willingness to Pay for Access to Greenspace

Given the financial constraints around GS provision outlined in the introduction, as well as criticism by community members directed at the use of GS by UDCs, this study explored UDCs willingness to pay for access. Around half (54%) of UDCs responded that they were in principle willing to pay for access, with highest support for payments of 10 (21%), 100 (14%), and 50 (12%) Japanese Yen per child per visit. In contrast, 28% were unwilling to pay and 18% declined to answer the question. Those UDCs unwilling to pay stated as reasons that payment was not financially viable, and placed the responsibility for GS provision with the municipality, prefecture and national government (financed through taxation). The role of parks as public space and public resources was emphasized, while some noted that UDCs already receive little to no support from the government (in comparison to other daycare facilities). Ideas for alternatives to support GS maintenance included volunteer activities (picking up litter, cleaning, weeding, planting, etc.) by the UDCs as well as other groups (elementary school students, senior volunteers etc.), an increase of public spending on GS, and membership systems based on monthly or yearly payment.

#### 3.1.5. Factors Influencing UDCs Provision of Greenspace Access

For off-premise GS use (Table 5), older UDCs seemed to provide more diverse and longer per-stay access to GS, while those with on-premise GS visited off-premise GS less frequently but sought out more diverse spaces. A higher number of children at the UDC correlated with encountering more conflict, but also more contact with GS managers or owners. No effects were found for the number of staff and licensing status of the UDCs. Similar to factors influencing GS access, prominent factors influencing caregivers’ perception of and willingness to pay for GS access included age of the facility, visit length, and diversity of GS used (Table 6). Overall, effects found were weak to moderate.

### 3.2. Parents Perspectives on Children’s Access to Greenspace

#### 3.2.1. Sample Characteristics and Choice of Daycare

A sample with responses of 3,645 parents from the 14 target cities with children under 5 in daycare was collected (Table 7). In Chiba and Kitakyushu, the target sample number (250) was not reached. Across cities, there was little fluctuation in mean age, but considerable variation in respondents’ educational attainment level and the percentage of those living in housing with GS. Among all parents, the three most frequent types of daycare used were kindergartens (44.8%), nursery schools (43.7%) and hybrid daycare centers (10%). In contrast, only 143 parents stated their children were visiting UDCs (hereafter called UDC parents in contrast to DC parents whose children attend licensed daycare centers). Likewise, 89% reported their daycare center to have outdoor play space. UDC parents were on average younger (d = 0.4), more highly educated (odds ratio (OR) = 1.52), more likely to be women (OR = 1.65) and live in shared housing without dedicated GS (x2(4) = 12.1, *p* < 0.05). For a majority of parents (54%) their daycare center was within one-kilometer distance of their home, and within one kilometer of a frequently used train station for one third. In contrast, for close to half (48%) their daycare center was more than two kilometers away from their workplace.

#### 3.2.2. Parents’ Reasons for Daycare Choice and Influencing Factors

**Figure 5:**
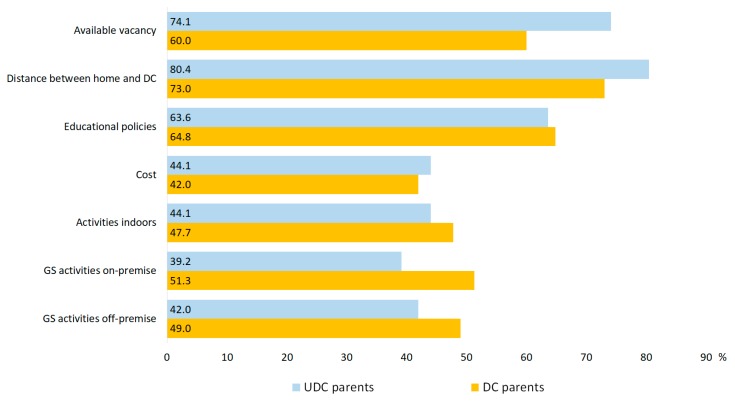
Parents’ reasons for choosing daycare centers. Percentage represents combined answers “important” and “very important”.

Overall, distance between home and daycare facility, educational policies, and available vacancies were parents’ top priorities in choosing daycare (Figure 5). UDC parents were more likely to prioritize distance between home and daycare facility (OR = 1.42) and available vacancies (OR = 2.2), but less likely to prioritize daycare centers for efforts to provide ample GS activities on-premise (OR = 0.62) as well as off-premise (0.72). Income was moderately negatively correlated with the importance of daycare cost (r = −0.145). Women expressed stronger preferences than men for all reasons except costs (d range: 0.11 to 0.20).

#### 3.2.3. Parents’ Perception of Children’s Access to Greenspace

When asked how frequently children in daycare were using off-premise GS, 10% of parents reported daily use, while an aggregate 33% reported weekly or more frequent use. In contrast, 9% reported their children never used off-premise GS. Only very few respondents (2.1%) had heard of trouble with managers or other users encountered by caregivers and children when using off-premises GS, and there was no significant difference in this number for UDC parents. Trouble parents had heard about was predominantly related to noise concerns, with some conflicts around elderly residents claiming space for gateball not allowing children to enter the GS.

**Figure 6:**
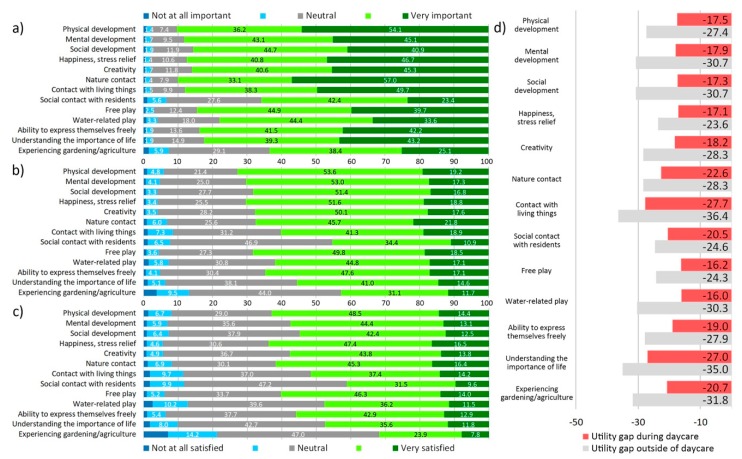
DC parents perception of GS: (**a**) importance of outdoor play for children aged five or under in regard to…, (**b**) satisfaction with greenspace regularly used during daycare in regard to…, (**c**) satisfaction with greenspace regularly used outside of daycare in regard to…, (**d**) utility gap of greenspace used during and outside of daycare (satisfaction-importance).

A large majority of DC parents (>80%) placed a high importance on the role of outdoor play for children’s development, with nature contact, physical development, and contact with living things being ranked as most important benefits (Figure 6). In contrast, less than 20% of parents were very satisfied with how well they perceived GS their children used during daycare to fulfil this role. The utility gap, which visualizes the discrepancies between parents’ expectations and satisfaction, was largest for contact with living things and understanding the importance of life, and exceeded 15 points for all items. For no item did regularly used GS fulfil or exceed parents’ expectations. 

**Figure 7:**
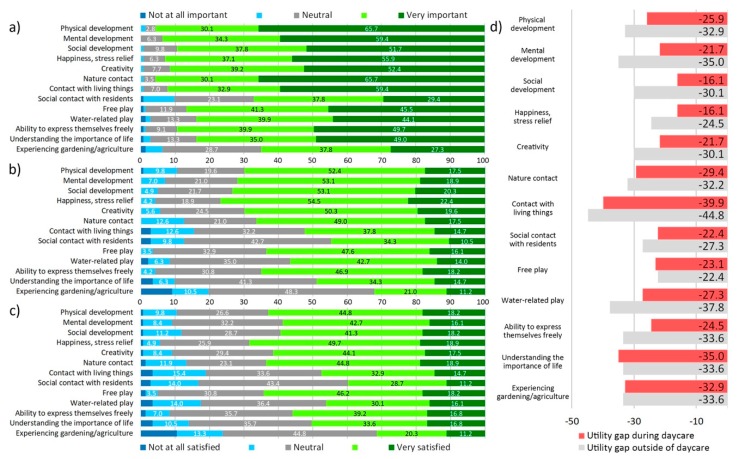
UDC parents perception of GS: (**a**) importance of outdoor play for children aged five or under in regard to…, (**b**) satisfaction with greenspace regularly used during daycare in regard to…, (**c**) satisfaction with greenspace regularly used outside of daycare in regard to…, (**d**) utility gap of greenspace used during and outside of daycare (satisfaction - importance).

A slightly different picture emerged when considering only UDC parents (Figure 7). While UDC parents did in general prioritize similar aspects of GS, they placed an even higher importance on the role of outdoor play (d = 0.22). This resulted in a slightly higher utility gap (d = 0.29), an effect that would appear even stronger with a utility gap calculation weighing “important” and “very important” responses differently.

When asked how frequently they used GS with their children outside of daycare, 5% of parents reported daily use, while an aggregate 37% reported weekly or more frequent use. In contrast, 6% reported their children never used off-premise GS. Parents reported that their satisfaction with children’s access to GS during daycare is closer to their expectations than they take their children to GS, resulting in a larger utility gap (Figure 6 and Figure 7).

#### 3.2.4. Parents’ Willingness to Pay for Children’s Access to Greenspace during Daycare

A large majority (92%) of parents were willing to pay to facilitate their children’s access to GS during daycare. Highest support was for payments of 10 (39%), 50 (20%), and 100 (19%) Japanese Yen per child per visit. In contrast, 8% were unwilling to pay. Reasons why parents were not willing to pay included foremostly a resolute rejection of the idea of paying for something commonly considered public space, with parents placing the responsibility for park maintenance with the local and national government (through taxation). Other reasons included unfairness due to daycare without GS not necessarily being a result of parents’ choice, unfairness due to the maintenance being placed unilaterally on daycare centers and parents, costs already being included in the daycare fees and thus to be shouldered by the daycare centers, the economic burden involved (and already perceived as high for parents with children struggling with daycare costs), the perception that such a system would threaten children’s GS access, the irony of a gap in government rhetoric between treasuring children in an aging nation but failing to act accordingly, doubt about maintenance costs for parks with little or no actual greenery, and not seeing the need for using GS. The amount respondents were willing to pay was weakly correlated with higher income (r = 0.057), being male (d = 0.090) and of older age (r = 0.064), placing a higher importance on GS (r = 0.086), being more satisfied with GS use of children during daycare (r = 0.079) and outside of daycare (r = 0.066).

#### 3.2.5. Factors Associated with Parents’ Attitude towards Greenspace Access

A range of demographic and other factors were associated with how satisfied parents were with GS access during daycare, how large the utility gap observed was, and how much money parents were willing to pay for access (Table 8). However, overall effects found were weak.

## 4. Discussion

### 4.1. Caregivers as Mediators: Potential and Limits of UDCs for Providing Children’s Access to Greenspace

Children depend entirely on caregivers to provide access to GS during daycare. Caregivers are aware of this, and their perception of the importance of outdoor play for children explains why most UDCs strive to provide daily access. This was also reflected in UDCs’ willingness to pay for access if required. Yet results suggest that apart from sufficient trained staff, three factors mostly outside of the control of caregivers and UDCs are vital for meeting children’s GS needs during daycare: adequate GS, a child-friendly social and physical environment, and institutional support. 

Given that caregivers are intimately familiar with children and their developmental needs, the utility gap between caregivers’ expectations and satisfaction can be interpreted as an expert assessment. Against the background of strong scientific evidence for the importance of children’s access to GS for their development [2,3,4,5,6,7], the results of this study strongly suggest that currently available GS is insufficient to meet children’s needs. For one, high utility gap aspects such as the lack of opportunities for experiencing gardening and agriculture, for water-related play, and for contact with living things, suggest GS may cover basic needs but fail to provide more diverse environments. While there are likely underexplored options such as vacant lots, safety concerns have been shown to limit their potential, particularly for younger children [55]. Yet an average utility gap of over 30 points also indicates the possibility of systemic underprovision (see also Table 2), both qualitative and quantitative, and reported issues of crowding represent further evidence for this. This is particularly important when considering how this study confirmed the reliance of UDCs on public greenspace previously reported [45]. While beyond the scope of this paper, future research might investigate on a national scale whether this issue is further complicated by spatial patterns in park distribution and local availability of non-park GS.

Results also suggest that GS access depends on more than adequate GS. Concerns raised about traffic safety and noise complaints in the neighborhood, and about GS use by other visitors that can make these spaces unsafe or unwelcoming for children such as littering, smoking and hostile attitudes point toward the need for a child-friendly social and physical environment. Given that UDCs are partly existing to sidestep resistance against provision of new, licensed daycare facilities, this is unsurprising but raises questions about whose needs are prioritized in an aging society. GS access is thus an issue not only for park designers and urban planners, but calls for a broader response by policy makers.

In this context, the very low level of institutional support UDCs reported receiving raises concerns about the adequacy of government action to facilitate GS access. Providing park information or permission to use GS for special events are necessary but not sufficient services. Municipal governments arguably ought to contribute, if not lead, public health and planning efforts to assist their stakeholders, particularly those without the means to do so themselves, in accessing vital resources. Yet it seems that by deregulating daycare provisions to allow UDCs address a social demand for daycare, in itself an act that can be interpreted as a neoliberal outsourcing of government functions, an attempt is made to offload the responsibility of care to private actors. While past research has investigated these issues from the perspective of women [1,24], it is becoming clear how such policies directly and indirectly also affect children.

Caregivers navigate these realities by making-do and mediating on behalf of those in their care. Data for this study suggests many are proactive, building ties with the community and visibly contributing through activities such as litter collection, out of an understanding of the precarity of their position. Currently, DCs with a longer history seem to have an advantage by knowing better where to take the children to, and they might also enjoy higher social capital and skill in negotiation, hence the longer GS visit times. The limited contact UDCs reported having with GS owners and managers could point towards potential for increasing caregivers’ positive influence. However, concerns about limited staff numbers highlight that caregivers’ primary duty is to take care of the children, and their time to engage in building community support for their activities is likely limited. Moreover, responses to the question of financial contributions to GS maintenance revealed a tension in the position of caregivers: they know GS access is vital, and many are willing to pay if need be, yet many also emphasize its nature as a basic public health service that should be thus financed in a socialized way through taxes. Caregivers thus are witnesses of a slow roll-back of government services, and are highly aware that compensation for underfunded services has limits. How conflicts between UDCs and GS managers (as reported around the popularity of university campus spaces at Hokkaido University [35]) are perceived as issues of resource overuse rather than underfunding and underprovision indicates that discussions around austerity and environmental justice have yet to enter mainstream debate in Japan. Such discussions, however, may be crucial to mobilize political support, which is why the role of parents may be larger than it appears at first. 

### 4.2. The Role of Parents for Children’s Access to Greenspace

Parents as legal guardians are ultimately responsible for their children’s needs being met. Results suggest that in comparison to caregivers as professionals, parents in general place less, yet still high importance on outdoor play for the development of children aged five or under. On the other hand, parents’ priorities for choosing daycare highlight their constraints in making these choices: basic conditions such as distance to the home, availability and educational policies must be satisfied first before issues such as access to GS or cost are considered. It is thus unsurprising that our parent survey, similar to the UDC survey, revealed substantial utility gaps for all aspects of child development. The utility gap remained when considering GS parents visited together with children. This indicates that accessing high quality GS is not simply a matter of making better choices for UDCs, but points towards a lack in GS quality as a fundamental, underlying issue. 

Given that parents are not able to simply choose daycare with better GS access, their willingness to pay, comparatively higher than that of UDCs, may be understood as an attempt to compensate with payments where the option arises, while doing so for distance to the home or vacancy would require substantially higher financial commitments. However, parents voiced many of the same concerns about a pay-to-access model as the UDCs did. In particular, the mentioned high financial burden on households with children, as well as weak but significant effects of parental income on the importance of cost in choosing daycare and amount of donations parents were willing to pay, suggest that introducing pay-to-access models would open the door to environmental injustice in accessing basic needs for children. This would add to the financial injustice built-in in the system and identified by previous research [1].

What role, then, do parents play in children’s access during daycare? Currently this role appears to be limited. Our data did not reveal a strong negative influence on children’s affordances around outdoor play, possibly because responsibility for children’s safety during this time is transferred to caregivers. On the other hand, parents did not seem to place high demands on daycare facilities to provide better access, despite how important they think it is. In fact, parents’ low awareness of conflicts around access GS caregivers reported experiencing suggests that the standard methods of communication between caregivers and parents, a daily notebook exchange and face-to-face contact when dropping off or picking up children, may not provide enough or the right opportunities to discuss these matters. The striking impact of the initially mentioned blog post about lacking daycare [28], however, points towards a potential for parents to wield significant influence should they choose to become more politically involved.

### 4.3. Implications for Stakeholders, Public Health, and Urban Planning

#### 4.3.1. Implications for Daycare Centers and Parents as Stakeholders

This study has three basic implications for UDCs. First, UDCs should be aware that parents may not realize what constraints affect GS access. Raising parents’ awareness and enlisting them in building a better neighborhood for children could be an effective action to take, given that local administrations are sensitive to opinions voiced by their constituents. This can open opportunities for improvements in GS through small grants, while strengthening the position and social capital of caregivers. Second, UDCs should aim to create lines of communication with GS managers, as our data shows some proactive ones already do. This allows GS managers to keep UDCs in the loop should issues arise, but also enables UDCs to convey their legitimate needs and requests, in particular if GS managers know that the UDCs speak not only on behalf of the children but also their parents. Ideally, UDCs may be able to form ties that can lead to larger improvements, for example through inclusion in community workshops to redesign local parks. Finally, UDCs in our study have reported a number of working strategies that could be shared and developed into guidelines for best practices, possibly through a formal or informal network of collaborating UDCs. These include actively building community support through greeting neighbors on the way and at the GS, as well as practicing and expressing stewardship of place by participating in community cleaning activities. One important goal here would appear to be to further normalize UDC visits to GS, thereby establishing a custom that may prevent other users from denying access. UDCs could also suggest possible activities to GS managers, from communal planting of flowers to temporary exhibiting art produced by the children.

Implications for parents are similarly focused on improving communication and using their influence to build a more supportive community environment. Parents should be aware that their engagement in this issue contributes to their children’s development. Enquiring about UDCs practices around GS access, as well as bringing the topic up in conversations with caregivers, children and fellow parents, could highlight the importance of the issue and contribute to reinforcing positive practices. Likewise, voicing opinions to the GS managers and particularly the local administration is known as an effective strategy that requires little time or money. In some places, community groups involved with GS maintenance may provide additional partners to enhance GS access and quality. Finally, addressing systemic underfunding of parks likely requires political action. Local elections and candidates can be sensitive to community issues [56], and support for such infrastructure in elections may prevent subsequent reductions in maintenance budgets.

#### 4.3.2. Implications for Public Health

One major implication is the urgent need for renewed investments in GS as a public health policy intervention. Our results suggest that UDCs practices do indeed add to existing pressures on GS. This demand is likely to further increase as societal trends outlined in the introduction continue. Especially noteworthy in this regard is the recent push of the Japanese government to provide free daycare across certain age ranges [57]. In this context, it is important to note that UDCs are not the only daycare facilities using public GS. The survey confirmed results of previous studies about the use of off-premise GS by regular daycare facilities despite having dedicated on-premise outdoor play space (which may or may not be vegetated) [36,40,41,42,45,46]. Yet users upset about large groups of children are likely not aware that some of these children have no dedicated on-premise GS to rely on. A general increase in daycare demand will thus lead to higher demand for GS. Given the overwhelmingly strong evidence for the benefits of urban greening for health [2,58,59] as well as adaptation to climate change [60], local and regional governments should strongly invest in GS as a public health policy aimed (not only) at children.

A number of additional implications center around building a more supportive institutional and community environment for enabling children’s access to GS. In this regard, public health departments will likely need to integrate horizontally by collaborating with urban planning and traffic departments for improving outcomes beyond GS boundaries [42], for example by stronger traffic regulation and no-car zones. Future research in this area could explore win-win scenarios around sustainability and community health. To increase public support for such measures, public health policy might aim to raise parents’ awareness of the importance of GS access as well as identifying and showing that caregivers are front line workers that deserve recognition and support for their service to communities.

#### 4.3.3. Implications for Urban Planning

Our study suggests that urban planning across the sites has failed in providing diverse, high quality, accessible GS that fulfils caregivers’ and parents’ expectations of its contributions to children’s development. Parks were by far the most frequently used GS type, but they may not be enough to satisfy children’s needs. Future research should identify if parks are used because they are preferred (and if so, whether caregivers are aware of better options), or whether they are used out of necessity. Local governments and planners should urgently address this issue through communicating with daycare centers to understand their needs, and by providing ample, diverse, high quality GS, including but not limited to parks. While this task may appear daunting given the extremely low provision in some cities (Table 2), and the anticipated increase in GS demand on top of the existing unmet needs, the demographic trajectory of Japanese cities trends towards depopulation and thus suggests there is potential for greening through proactively using vacant lots [55,61,62]. Cities have yet to make use of this potential, but there are ways for local administrations to work with residents towards participatory management models. Previous research in fact highlights that there is a strong mandate for investment into GS, but also indicates that simply pushing the responsibility for maintenance on residents without providing necessary support is not well received by residents and may lead to negative outcomes [25]. 

In light of this need for a renewed focus on planning urban GS, our study identified an important issue around the spatial organization of GS. Classic Japanese greening plans aim at providing GS within walking distance from residents’ homes [63]. While our results show that these parks are indeed important, the location of daycare centers near other focal points of parents’ and children’s lives, such as public transport nodes and parents’ workplaces, can lead to new GS demand ‘hot spots’. Further research is thus required to analyze how well current GS serve these areas and where potential GS demand hot spots might be that require being addressed to provide better access. As noted above, planners may also need to consider that standard parks appear to fail children’s needs for diverse environments. Thinking beyond GS boundaries, it is also necessary to establish safe travel routes to and from daycare centers to GS. As noted in the previous section, this may require an increase in no-car zones, an urban feature still comparatively rare in Japanese cities.

### 4.4. Limitations of the Study

This study has a number of limitations that need to be considered when interpreting the results. Firstly, the study is limited to Japan. Research from Chinese cities suggests however that the issues identified herein may not be unique to Japan, with problems such as gaps between needs and reality in natural playground planning and design as well as lack of outdoor play time [64,65,66]. Future research might compare children’s access to greenspace between countries with similar circumstances such as high urban density and aging populations, applying an environmental justice lens for analysis. Within Japan, the study covers a wide range of Japanese cities, but it did not cover all major cities or more rural areas. The response rate for the UDC survey was low despite reminder postcards and other measures taken (transparent postal envelope, use of the home institution’s mascot to appeal to respondents). One reason may be that recent years have seen a general decline in the response rate for surveys [67]. Additionally, the fluctuating nature of UDCs and the time constraints experienced by caregivers that may not allow them to participate in a voluntary, non-compensated survey are likely factors contributing to a low response rate. As such, the UDCs represented in the survey may be biased towards those with better a better staff situation. Unfortunately, follow-up telephone reminders were not feasible due to their cost. Similar limitations apply to the sample of parents of children visiting UDCs. Another limitation of the study is the degree to which respondents can be guaranteed to share an understanding of the concepts in the survey instruments (e.g., physical development). However, this is likely to be mitigated by the shared professional background of caregivers, and the larger sample size of parents. This study did not include multivariate regression analysis, but rather focused on exploring a variety of dependent variables. Future research should consider devising a multivariate model in addition to follow-up qualitative research, for example in the form of interviews, to complement the quantitative focus of this study, both for UDCs and for parents. This study did also not directly ask children about their experience with GS during daycare, a topic also requiring further research. 

## 5. Conclusions

This study investigated what role caregivers and parents play for children’s GS access during their stay in unlicensed daycare centers, which often lack on-premise outdoor play spaces. Results showed that caregivers proactively strive to provide access, yet see their efforts constrained by GS that does not reflect the importance for children’s development caregivers perceive, hostile behavior by neighbors and other park users, and a lack of institutional support. Caregiver’s support for a pay-to-access model was moderate and placed in the context of environmental justice concerns. In comparison, parents’ role appears to be limited, with GS not ranking high among parents’ priorities when selecting daycare providers. Parents also showed little awareness of issues caregivers encounter in providing GS access. In summary, children’s access to GS during daycare is thus threatened predominantly by a qualitative and quantitative lack of available GS, a child-friendly social and physical environment, and institutional support for caregivers. Limitations included the geographical extent of the survey, a low UDC response rate, and a focus on quantitative methods.

This study has implications for caregivers, parents, public health policy and urban planning. Caregivers and parents would likely benefit from increased communication and collaboration to support GS access through placing better GS and institutional support firmly on the agenda of local administrations. From a public health perspective, the importance of addressing long-standing systemic underfunding and underprovision of services by improving GS quality and quantity to meet children’s GS needs is very clear. This importance is only increased in the context necessary adaptation to climate change. Likewise, local governments and urban planners need to face the failure of past policies and planning in providing adequate GS, and urgently act to address the issue, because ongoing social trends and the national government policy shift to free daycare will likely result in further increased demand for GS.

## Figures and Tables

**Table 1:** Major types of daycare centers in Japan and their characteristics (adapted from [34]).

Characteristics	Licensed Daycare Centers				Tokyo Metropolitan Government Certified Hoikusho		Unlicensed Daycare Centers
**Subtypes**	*Hoikusho*		*Kodomo-en*	*Yochi-en*	Type A	Type B	Baby hotel, nursery center ^2^ etc.
**Ministry in charge ^1^**	MHLW		MHLW, MEXT	MEXT	Bureau of Social Welfare and Public Health (Tokyo)		Local government
**Legal & regulatory basis**	Child Welfare Law		Preschool Childcare Promotion Act ^3^	School Education Law	Tokyo Metropolitan Government certified *hoikusho* implementation guidelines		Unlicensed Daycare center supervision standard, Child Welfare Law
**Catered age**	Birth to start of primary school			From age 3	Birth to start of primary school	Birth to 2 years old	Birth to start of primary school
**Daycare hours**	8 h			4 h	13 h		No regulation
**Size**	>60		>35 per grade	>35 per grade	20~120 (half of children should be aged less than 3)	6~29	No regulation
**Required facilities**	Daycare room, playroom, infant/crawling room, restroom, kitchen, dispensary			Daycare room, playroom, restroom, dispensary, drinking water, washing facilities, staff room	Daycare room, kitchen, restroom, dispensary, etc.		Daycare room, kitchen, restroom
**Maximum child to caregivers ratio**	0 years	3:1	3:1		3:1		3:1
	1 or 2 years	6:1	6:1		6:1		6:1
	3 years	20:1	35:1 short time 20:1 long time	Min. 1 teacher per 1 grade (<35)	20:1		20:1
	4+ years	30:1	35:1 short time 30:1 long time		30:1		30:1
**Minimum daycare room area**	0–1 years	Daycare room: 1.65 m^2^ per child; Infant/crawling room: 3.3 m^2^ per child			3.3 m^2^ per child	2.5 m^2^ per child	1.65 m^2^ per child
	2+ years	1.98 m^2^ per child		1+ room per grade	1.98 m^2^ per child		
**Minimum playground area**	3.3 m^2^ per child aged 2+ (can be substituted by nearby GS)		3.3 m^2^ per child aged 2; refer to *Yochi-en* standard for 3+ years	2nd + lower grades: 330 + 100 × (grade-1) m^2^; 3rd + higher grades: 400+80 × (grade-3) m^2^	3.3 m^2^ per child aged 2+ (can be substituted by nearby GS)	Not required	Not required

^1^ Ministry abbreviations: MHLW (Ministry of Health, Labour and Welfare), MEXT (Ministry of Education, Culture, Sports Science and Technology); ^2^ in workplaces, hospitals etc.; ^3^ Full name: Act on Advancement of Comprehensive Service Related to Education, Child Care, etc. of Preschool Children.

**Table 2:** Overview of study site characteristics [48,49,50,51].

Study Site	Population (2015)	Projected Population (2045)	Population Density (2015)	Green Space Per Capita (m^2^) (2017)	Number of UDCs (2017)
Tokyo	13,515,271	13,606,683	6168.7	3.0	1300
Yokohama	3,724,844	3,446,124	8514.1	4.9	281
Osaka	2,691,185	2,410,820	11,949.7	3.5	157
Nagoya	2,295,638	2,173,770	7032.1	7.0	108
Sapporo	1,952,356	1,805,120	1741.2	12.6	89
Fukuoka	1,538,681	1,654,572	2759.8	8.4	135
Kobe	1,537,272	1,295,786	4480.9	17.5	63
Kawasaki	1,475,213	1,549,981	1782.0	3.8	173
Kyoto	1,475,183	1,297,241	10,316.2	4.4	37
Saitama	1,263,979	1,285,867	5813.3	5.1	144
Hiroshima	1,194,034	1,122,112	1317.1	7.8	72
Sendai	1,082,159	922,655	1376.3	15.2	81
Chiba	971,882	905,240	3576.3	9.6	38
Kitakyushu	961,286	771,168	1954.0	12.4	54

**Table 3:** Characteristics of surveyed unlicensed daycare centers.

City	N	Range of Founding Year	Range of Staff No.	Range of Child. No.	Average Staff No.	Average Children No.	Average Student Teacher Ratio	Average Care Time	% With GS
Tokyo	74	1949–2018	1–60	2–289	10.6	33.4	3.5	8.5	34.7
Yokohama	13	1983–2015	4–20	6–69	9.1	31.1	4.1	9.3	23.1
Osaka	6	1999–2017	2–15	5–50	7.0	18.7	4.6	8.3	0.0
Nagoya	9	1924–2016	2–23	8–200	9.0	45.3	4.5	7.3	44.4
Sapporo	7	2002–2017	3–17	10–36	8.8	22.3	2.7	9.7	50.0
Fukuoka	12	1992–2016	4–12	18–84	7.3	34.3	5.5	9.6	41.7
Kobe	6	2003–2017	3–35	14–25	13.7	19.4	3.2	8.5	50.0
Kawasaki	14	1993–2015	4–25	15–89	13.0	33.8	3.2	10.0	14.3
Kyoto	2	2012–2015	3–8	6–30	5.5	18	3.08	7.75	0
Saitama	8	1969–2018	3–26	10–72	14.0	39.6	2.9	10.1	62.5
Hiroshima	1	2013	4	50	4.0	50.0	12.5	10.0	0.0
Sendai	5	2003–2017	4–13	10–72	7.8	33.2	4.0	7.9	20.0
Chiba	2	1994–2001	5	23–28	5.0	26.5	3.5	9.5	0
Kitakyushu	10	1996–2017	3–18	9–54	6.3	25.8	4.9	8.1	50.0
Total	169 + 4 ^1^								

^1^ Total number includes 4 UDCs with unknown location.

**Table 4:** Overview of UDC practices around GS use.

Practice	Frequency/Time/Type	UDCs (%)
Frequency of visiting green spaces	Never	2.3
	Less than once a month	3.5
	2~3 times a month	6.4
	Once a week	4.6
	2~3 times a week	19.7
	Everyday	63.0
	N/A	0.6
Frequency of visiting other forms of open spaces (roadsides, commercial settings, etc.)	Never	61.8
	Less than once a month	9.2
	2~3 times a month	7.5
	Once a week	4.0
	2~3 times a week	6.4
	Everyday	6.9
	N/A	4.0
Staying time in green space	Less than 30 min	6.4
	0.5~1 h	63.6
	1~2 h	16.8
	2~3 h	5.8
	More than 3 h	1.7
	N/A	5.8
Types of green space	Parks	96.0
	Riversides	19.1
	University campuses	2.3
	Temples and shrines	24.3
	Farms	11.0
	Forests	5.2
	Vacant lots	4.0
	Others	23.7
	N/A	1.7

**Table 5:** Significant effects of UDC attributes on off-premise GS use (*p* < 0.05).

UDC Attributes	Visit Frequency	Visit Length	Diversity of Spaces Used	Conflict Encountered	Communication with GS Manager/Owner
Age of facility	X	older = slightly longer	older = slightly higher ^1^	X	X
Number of staff	X	X	X	X	X
Number of children	X	X	X	Medium increase	Small increase
Certification	X	X	X	X	X
On-premise GS	lower	X	Higher ^2^	X	X

X = no significant effect; ^1^ Older DCs are more likely to visit agricultural land.; ^2^ DCs owning GS are more likely to visit Forest and Vacant land.

**Table 6:** Significant effects of UDC attributes on caregivers’ perception of and willingness to pay for GS access (*p* < 0.05).

Affected Item	Attribute	Effect Size	Interpretation
Overall expectations for GS	Age of facility	0.163 ^1^	Newer UDCs were slightly more likely to have higher expectations
	Number of children	−0.151 ^1^	UDCs with fewer children were slightly more likely to have higher expectations
	Visit length	0.149 ^1^	Longer visits were weakly correlated with higher expectations
Satisfaction with regularly used GS	Age of facility	−0.185 ^1^	Newer UDCs were slightly more likely to be less satisfied
	Visit length	0.243 ^1^	Longer visits were weakly correlated with higher satisfaction
	Diversity of GS used	0.153 ^1^	Higher diversity were weakly correlated with higher satisfaction
	On-premise GS	0.374 ^2^	UDCs with GS were moderately more likely to be more satisfied
Utility gap between expectations and satisfaction	Age of facility	−0.261 ^1^	Newer UDCs were slightly more likely to have a larger utility gap
	On-premise GS	0.373 ^2^	UDCs with GS were moderately more likely to have a smaller utility gap
	Visit length	0.251 ^1^	Longer visits were weakly correlated with a smaller utility gap
	Diversity of GS used	−0.177 ^1^	Lower diversity was weakly correlated with a larger utility gap
Willingness to pay	Staff number	0.445 ^2^	Lower staff number was moderately correlated with higher willingness to pay
	Opening hours	0.246 ^2^	Shorter opening hours were weakly correlated with higher willingness to pay
	Diversity of GS used	−0.414 ^2^	Lower diversity was moderately correlated with less willingness to pay
Donation degree	Age of facility	−0.297 ^1^	Newer UDCs were slightly more likely to be willing to pay a smaller amount
	Diversity of GS used	0.211 ^1^	Higher diversity was weakly correlated with willingness to pay a larger amount

^1^ Spearman’s r; ^2^ Cohen’s d.

**Table 7:** Characteristics of surveyed parents.

Study Site	Number of Samples (N)	Parents with Children in UDCs (%)	Education Undergraduate or Higher (%)	Housing with GS (%)	Mean Age
Tokyo	500	2.8	84.4	29.8	39.6
Yokohama	250	2.8	82.8	46.4	38.9
Osaka	250	6.0	72.8	24.0	38.3
Nagoya	250	2.8	83.2	43.2	37.8
Sapporo	250	5.6	67.6	46.0	37.8
Fukuoka	250	4.2	74.0	30.8	37.4
Kobe	250	4.8	75.6	50.0	37.6
Kawasaki	250	4.4	85.6	38.4	38
Kyoto	250	3.6	77.6	33.2	37.9
Saitama	250	4.4	83.2	48.8	38
Hiroshima	250	3.6	79.2	44.4	37.1
Sendai	250	4.8	71.2	49.6	37.1
Chiba	206	1.0	83.0	62.6	38.4
Kitakyushu	189	3.7	64.6	46.6	37.1

**Table 8:** Significant effects of factors influencing parents’ perception of and amount willing to pay for green space access (*p* < 0.05).

Aspect of Perception	Factors	Effect Size	Interpretation
Satisfaction with green space access during daycare	City	0.013 ^1^	Parents from Tokyo, Osaka and Nagoya are less satisfied; Parents from Sapporo, Kitakyushu, and Kyoto are more satisfied
	Education	−0.05 ^2^	Educational attainment was weakly correlated with lower satisfaction
	Income	−0.037 ^2^	Income was weakly correlated with lower satisfaction
	Frequency of GS visits by caregivers	0.039 ^2^	Lower frequency was weakly correlated with lower satisfaction
	Gender	−0.229 ^3^	Men were slightly less likely to be satisfied
	Age	−0.057 ^2^	Age was weakly correlated with lower satisfaction
	Having GS in facilities	0.219 ^3^	Parents with children in daycare without on-premise GS were less likely to be satisfied
Utility gap	Gender	0.146 ^3^	Men had on average a larger utility gap
	Having GS in facilities	0.159 ^3^	Parents of children in daycare centers without on-premise GS had a larger utility gap
	License	0.288 ^3^	Unlicensed daycare centers had a larger utility gap
	Frequency of GS visits by caregivers	0.039 ^2^	Frequency was weakly correlated with a smaller utility gap
Amount of donation	Income	0.057 ^2^	Income was weakly correlated with higher donation amounts
	Frequency of GS visiting by care-givers	−0.068 ^2^	Frequency was weakly correlated with lower donation amounts
	Gender	7.61 ^1^	Women reported on average lower donation amounts
	Having GS in facilities	8.57 ^1^	Parents of children in daycare centers without on-premise GS reported on average lower donation amounts

^1^ Ɛ^2^, ^2^ Spearman’s r, ^3^ Cohen’s d; Parents are generally supportive of donation.

## Supplementary Materials

The following are available online at https://www.mdpi.com/1660-4601/17/6/1948/s1, File S1: Unlicensed daycare center survey instrument (in Japanese); File S2: Parent survey instrument (in Japanese); File S3: Unlicensed daycare center survey data set (in Japanese); File S4: Parent survey data set (in Japanese).
