# Supplementary material for: Understanding Threats to Young Children’s Green Space Access in Unlicensed Daycare Centers in Japan
Source: Int J Environ Res Public Health. 2020 Mar 16;17(6):1948. doi: 10.3390/ijerph17061948 (PMC7143442; doi:10.3390/ijerph17061948)

大学共同利用機関法人　人間文化研究機構
総合地球環境学研究所
〒603-8047　京都市北区上賀茂本山457番地4
Tel.075-707-2100（代）　Fax.075-707-2106（代）
http://www.chikyu.ac.jp/

Inter-University Research Institute Corporation National Institutes for the Humanities
Research Institute for Humanity and Nature
457-4 Motoyama, Kamigamo, Kita-ku, Kyoto, 603-8047, JAPAN
Tel. +81-75-707-2100　Fax. +81-75-707-2106

保育施設様　各位　　　　　　　　　　　　　　　　　　平成 30 年 6 月

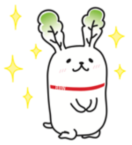

子どもの外遊びアンケートご協力のお願い

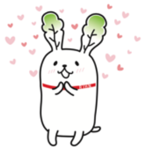

時下ますますご清祥のこととお慶び申し上げます。
この度は、文部科学省の科学研究費助成事業の研究の一環として、保育施設で行われる外遊びについてのアンケート調査を行なっております。保育施設定員数が注目される中で、保育施設で行われる外遊びが子どもたちへどのような役割や課題を持っているのかを明らかにすることを目的としています。そのため、保育施設と子どもの外遊びについて皆さまにお尋ねしています。このアンケートで、「外遊び」は「緑にふれる機会がある」園外保育活動のみとしています。
皆さまのご協力をお願い申し上げます。

ご協力いただきたいこと

　子供の外遊びアンケートにご記入のうえ、同封の返信用封筒にアンケート用紙を入れてご返送ください。ご回答は **7 月末まで**にご返送をお願いいたします。

参加への同意方法・プライバシーの保護について

　本アンケート調査は完全に任意です。回答中にいつでも中断・中止することができ、どの質問に対しても、答えることを拒否する自由があります。個人情報は集めません。保育施設を特定できる情報を公表することはありません。アンケートにお答えいただくことをもって、調査協力にご承諾いただいたことといたします。回収管理のため、アンケートには発送番号を記載させていただきます。ご不明な点は、調査担当者へお問い合わせください。

調査結果の公表やご不明な点がある場合のお問い合わせについて

　結果は 2019 年度までに研究学術誌に発表する予定です。調査結果を知りたい方や質問がある方は 2018 年 9 月末までに下記にご連絡ください。

調査担当者：　[name, email]
調査委託先（返送先）：　[company name, address]

子どもの外遊びアンケート

大学共同利用機関法人　人間文化研究機構
総合地球環境学研究所

このアンケートは、文部科学省の科学研究費助成事業の研究の一環として行なっているものです。保育施設定員数が注目される中で、保育施設で行われる外遊びが子どもたちへどのような役割や課題を持っているのかを明らかにすることを目的としています。そのため、保育施設と子どもの外遊びについて皆さまにお尋ねしています。このアンケートで、「外遊び」は「緑にふれる機会がある」園外保育活動のみとしています。皆さまのご協力をお願い申し上げます。

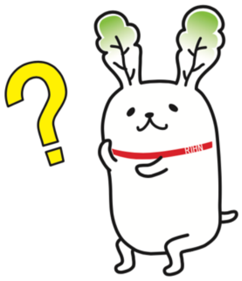

ご協力いただきたいこと

　アンケートにご記入のうえ、同封の返信用封筒にアンケート用紙を入れてご返信ください。ご回答は 7 月末までにご返送をお願いいたします。

該当する項目に、✔を入れてください！

保育施設についてお聞かせください			
Q1. 設立年	＿＿＿＿＿＿　（西暦）	Q2. 職員数	＿＿＿＿＿＿　人
Q3. 定員	＿＿＿＿＿＿　人	Q4. 認証書	□有り　□無し
Q5. 預かり時間（平均）	＿＿＿＿＿＿　時間	Q6. 施設内の緑地（芝生や木等がある）	□有り　□無し

施設外の緑地利用についてお聞かせください
Q7. 子どもたちの外遊びの際、施設外の緑地（例：近所の公園、川沿い・河川敷、 農園、大学キャンパス内の緑地、お寺・神社、森、空き地、他の公共・私有緑地など）をどんな頻度で利用しますか？<br>□利用しない　□月に１回以下　□月に２～３回　□週に１回　□週に２～３回　□ほぼ毎日
Q8. 施設外で、どのような緑地を利用しますか？当てはまる全てに✔を入れてください。<br>□近所の公園　□川沿い・河川敷　□大学内の緑地　□お寺・神社　□ 農園　□森　□空き地<br>□他の公共緑地・私有緑地（＿＿＿＿＿＿＿＿＿＿＿＿＿＿＿＿）
Q9. 緑地以外に、道路・商店街等を施設外の外遊びの場として、どんな頻度で利用しますか？<br>□利用しない　□月に１回程度　□月に２～３回　□週に１回　□週に２～３回　□ほぼ毎日
Q10. 施設外の緑地を利用する時、利用時間はどれくらいですか？<br>□30 分以下　□30 分～１時間　□１～２時間　□２～３時間　□３時間以上

Q11. 利用する緑地の名前を教えてください。３箇所以上を利用する場合は、利用する頻度の高い３つの緑地の名前に丸をつけてください。

________________　________________　________________

________________　________________　________________

________________　________________　________________

施設外の緑地利用に関する方針、仕組み、トラブルや課題についてお聞かせください

Q12. 施設を設立した時、外遊びについてどのような計画がありましたか？（例：1 日の活動の中で外遊びの取り入れ方 、利用する緑地の特定、自治体と外遊びについての相談など）

Q13. 設立時と比べ、外遊びの活動などに変更ありましたか？あった場合、そのきっかけは何でしたか？

□変更はなかった

□変更があった：

Q14. 子どもたちの外遊びをよりよくするために、一番やるべきことは何だと思いますか？

Q15. 利用している緑地の管理人・所有者などと、利用に関する話し合いをされたことはありますか？

□話したことはない　　□話したことがある（内容：________________）

Q16. 緑地を利用する時、利用に支障をきたすような、管理人や他の利用者や近隣住民とのトラブルはありましたか？（例：子どもの遊び声に対する苦情、冷たい目で見られるなど）
Q17. 遊びのための緑地を確保することに対して、自治体などの支援を受けていますか？（例：適切な緑地の地図や情報の提供、私有地の緑地所有者の交渉のサポートなど）
Q18. 利用者が多い場合、緑地の整備費（芝生管理など）が高くなることがあります。もし利用している緑地の整備のために寄付制度が設立されることになったら、実際に出すとするならばどの程度の定期的な寄付金が手頃だと思いますか？（子ども１人当たり・利用１回当たり）<br><br>☐寄付しない　☐１０円　☐２０円　☐３０円　☐５０円　☐ １００円
Q19. 上の質問で「寄付しない」と答えた場合、寄付しない・できない理由を教えてください：
Q20. 寄付制度以外、共同で利用する緑地の整備を支援できるアイデアがあれば教えてください

次のページで最後です☞

Q21. 子どもたち(５歳以下)のために、外遊びは以下の点でどれくらい重要だと思いますか？

当てはまる番号に〇をつけてください	**全く重要でない** ←				→ **非常に重要**
1. 身体的発達・運動能力	1	2	3	4	5
2. 精神的発達・認知発達	1	2	3	4	5
3. 社会的発達	1	2	3	4	5
4. 幸福・ストレス緩和	1	2	3	4	5
5. 想像力	1	2	3	4	5
6. 自然にふれる	1	2	3	4	5
7. 生き物にふれる	1	2	3	4	5
8. 地域の人々との交流	1	2	3	4	5
9. 自由遊び	1	2	3	4	5
10. 水遊び	1	2	3	4	5
11. 自分を自由に表現できる能力	1	2	3	4	5
12. 命の大事さを知る	1	2	3	4	5
13. 農業体験	1	2	3	4	5
14. その他に緑地遊びの重要な意義があれば、教えてください：					

Q22. よく利用する緑地の遊びで、以下の点についてどれくらい満足していますか？

当てはまる番号に〇をつけてください	**全く満足しない** ←				→ **非常に満足**
1. 身体的発達・運動能力	1	2	3	4	5
2. 精神的発達・認知発達	1	2	3	4	5
3. 社会的発達	1	2	3	4	5
4. 幸福・ストレス緩和	1	2	3	4	5
5. 想像力	1	2	3	4	5
6. 自然とふれる	1	2	3	4	5
7. 生き物とふれる	1	2	3	4	5
8. 地域の人々との交流	1	2	3	4	5
9. 自由遊び	1	2	3	4	5
10. 水遊び	1	2	3	4	5
11. 自分を自由に表現できる能力	1	2	3	4	5
12. 命の大事さを知る	1	2	3	4	5
13. 農業体験	1	2	3	4	5

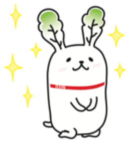

長時間のご協力、誠にありがとうございました！

同封の返信用封筒にアンケート用紙を入れてご返信ください。

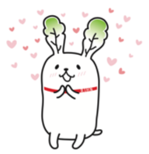

Supplement: Supplementary file 1 [file ijerph-17-01948-s001.zip › File S1 UDC survey instrument.pdf]
