# Supplementary material for: Understanding Threats to Young Children’s Green Space Access in Unlicensed Daycare Centers in Japan
Source: Int J Environ Res Public Health. 2020 Mar 16;17(6):1948. doi: 10.3390/ijerph17061948 (PMC7143442; doi:10.3390/ijerph17061948)
Supplement: Supplementary file 1 [file ijerph-17-01948-s001.zip › File S2 Parent survey instrument.pdf]

「保育施設に関するアンケート」一覧プレビュー

簡易表示 条件設定閉

本システムはパソコンの推奨動作環境をWindows OSのみとしております。それ以外のOSの場合、正常に動作しない可能性があります。

保育施設に関するアンケート

モニターの皆様へのお願い

本アンケートには、一般に公開していない情報が含まれる場合があります。
アンケート内で知り得た情報について、決して第三者に口外しないよう、お願いします。

「第三者への口外」に含まれる例

口頭、電話、メール等で友人・知人に話す

SNSやブログ、掲示板等に書き込む

その他、手段を問わず、情報を第三者に伝達する行為

注意事項

複数のアンケート画面を同時に開くと、正常に回答できません。
アンケートはひとつずつ、回答ください。

アンケートへの回答は、「動作環境」に記載の環境からお願いします。

本アンケートは、回答を中断してから1時間以内は中断した質問から再開可能です。
（システム緊急対応等により再開できない場合もありますので、予めご了承ください。）

回答結果は、当社の「個人情報保護方針」に基づいて取り扱います。

上記の内容をご確認いただき、同意してご協力いただける場合のみ、「同意し、アンケート開始」を押してアンケートを開始してください。

同意し、アンケート開始

改ページ

SC1
必須
あなたの居住地域を教えてください。

1.札幌市
2.仙台市
3.東京23区
4.横浜市
5.さいたま市
6.川崎市
7.千葉市
8.大阪市
9.名古屋市
10.神戸市
11.京都市
12.広島市
13.福岡市
14.北九州市
15.上記以外

次へ

終了条件

SC

優先順位	条件名	条件式
1	調査対象外のため	(SC1 **or** 15)

改ページ

SC2
必須
現在、同居している「就学前の」お子さますべての本年度の年齢をお知らせください。（いくつでも）

1.0歳（2019年4月2日以降に生まれた）
2.1歳（2018年4月2日～2019年4月1日生まれ）
3.2歳（2017年4月2日～2018年4月1日生まれ）
4.3歳（2016年4月2日～2017年4月1日生まれ）
5.4歳（2015年4月2日～2016年4月1日生まれ）
6.5歳（2014年4月2日～2015年4月1日生まれ）
7.6歳（2013年4月2日～2014年4月1日生まれ）
8.子どもはいない／同居している子どもはいない／就学前の子どもはいない**(排他)**

次へ

[選択肢] 番号は回答者へは表示しません

改ページ

条件設定

選択肢表示条件

前提条件　全て表示

No	条件式	表示項目
1	(SC1 **ornot** 3~7)	1. 保育園<br>2. 幼稚園<br>3. 認定こども園（幼保一体型）<br>6. 無認可保育園・認可外保育園（企業内・院内保育所・託児所）<br>7. 無認可保育園・認可外保育園（ベビーホテル）<br>8. 無認可保育園・認可外保育園（その他）<br>9. その他<br>10. 保育施設には通っていない

質問表示条件

条件式
(SC2 **or** 1~7)

SC3　お子さまはどのような保育施設に通っていますか。
必須　（いくつでも）

1.保育園

2.幼稚園

3.認定こども園（幼保一体型）

4.認証保育所A型（東京、0歳から小学校まで可能）

5.認証保育所B型（東京、0歳から2歳まで可能）

6.無認可保育園・認可外保育園（企業内・院内保育所・託児所）

7.無認可保育園・認可外保育園（ベビーホテル）

8.無認可保育園・認可外保育園（その他）

9.その他

10.保育施設には通っていない　**(排他)**

次へ

SC終了

終了条件

SC

優先順位	条件名	条件式
1	調査対象外のため	(SC1 **or** 15)

クォータ設定

SC終了質問

No	項番	質問	改ページ	質問タイプ
1	SC3	お子さまはどのような保育施設に通っていますか。（いくつでも）	改ページ	チェックボックス

本調査対象条件

優先順回収

No	条件名	条件式
1	札幌市	((SC1 **or** 1) **AND** (SC3 **or** 1~8))
2	仙台市	((SC1 **or** 2) **AND** (SC3 **or** 1~8))
3	東京23区	((SC1 **or** 3) **AND** (SC3 **or** 1~8))
4	横浜市	((SC1 **or** 4) **AND** (SC3 **or** 1~8))
5	さいたま市	((SC1 **or** 5) **AND** (SC3 **or** 1~8))
6	川崎市	((SC1 **or** 6) **AND** (SC3 **or** 1~8))
7	千葉市	((SC1 **or** 7) **AND** (SC3 **or** 1~8))
8	大阪市	((SC1 **or** 8) **AND** (SC3 **or** 1~8))
9	名古屋市	((SC1 **or** 9) **AND** (SC3 **or** 1~8))
10	神戸市	((SC1 **or** 10) **AND** (SC3 **or** 1~8))
11	京都市	((SC1 **or** 11) **AND** (SC3 **or** 1~8))
12	広島市	((SC1 **or** 12) **AND** (SC3 **or** 1~8))
13	福岡市	((SC1 **or** 13) **AND** (SC3 **or** 1~8))
14	北九州市	((SC1 **or** 14) **AND** (SC3 **or** 1~8))

本調査開始

■このアンケートでは、保育施設と子どもの外遊びについてお伺いします。

保育施設で行われる外遊びが子どもたちへどのような役割を担い、また課題をもたらすのかを明らかにすることを目的としています。

このアンケートで、「外遊び」は「緑にふれる機会がある」園外保育活動のみとしています。ご協力をお願い申し上げます。

あなたご自身についてお伺いします。

Q1 必須 どのような家に住んでいますか。

1.庭がある住宅（一戸建て等）

2.庭がない住宅（一戸建て等）

3.共同利用緑地（庭など）があるアパート・マンション等

4.共同利用緑地（庭など）がないアパート・マンション等

5.その他

次へ

改ページ

Q2 必須 最終学歴を教えてください。

1.中卒

2.高卒

3.大学卒（学士・短大卒・専門大卒等）

4.大学院卒（修士・博士等）

5.回答したくない

次へ

改ページ

Q3 必須 年間世帯収入はどれくらいですか。

1.200万円未満

2.200万円～400万円未満

3.400万円～600万円未満

4.600万円～800万円未満

5.800万円～1,000万円未満

6.1,000万円～1,250万円未満

7.1,250万円以上

8.回答したくない

次へ

[選択肢] 番号は回答者へは表示しません

改ページ

Q4 必須 お住まいの郵便番号を教えてください。

郵便番号 必須 000 - 0000 ＜半角数字＞

次へ

改ページ

Q5
必須

お子さまが通っている保育施設についてお伺いします（お子さまが何人かいる場合、5歳以下の一番上のお子さまについて教えてください）。
お子さまが通っている保育施設を選んだ理由のなかで、以下の点はどの程度重要でしたか。

※この設問は、それぞれ横方向（→）にお答えください。

	1.全く重要でない	2.あまり重要でない	3.どちらでもない	4.重要	5.非常に重要
1. 空きがあった					
2. 自宅・勤め先・通勤などによく使う駅に近い					
3. 教育方針が気に入った					
4. 値段が妥当					
5. 屋内の遊びが充実してそうだった					
6. 施設「内」の緑地での外遊び活動が充実してそうだった					
7. 施設「外」の緑地での外遊び活動が充実してそうだった					

次へ

改ページ

Q6
必須

お子さまが通っている保育施設とその距離についてそれぞれ当てはまるものを選んでください。

自宅

1.自宅（保育施設から約1キロ未満、大人が歩いて15分程度）

2.自宅（保育施設から約1キロ以上から2キロ未満、車で5分程度）

3.自宅（保育施設から約2キロ以上、車で5分以上、大人が歩いて30分以上）

勤め先

4.勤め先（保育施設から約1キロ未満、大人が歩いて15分程度）

5.勤め先（保育施設から約1キロ以上から2キロ未満、車で5分程度）

6.勤め先（保育施設から約2キロ以上、車で5分以上、大人が歩いて30分以上）

通勤などによく使う駅

7.通勤などによく使う駅（保育施設から約1キロ未満、大人が歩いて15分程度）

8.通勤などによく使う駅（保育施設から約1キロ以上から2キロ未満、車で5分程度）

9.通勤などによく使う駅（保育施設から約2キロ以上、車で5分以上、大人が歩いて30分以上）

その他

10.その他（保育施設から約1キロ未満（大人が歩いて15分程度））

11.その他（保育施設から約1キロ以上から2キロ未満、車で5分程度）

12.その他（保育施設から約2キロ以上、車で5分以上、大人が歩いて30分以上）

次へ

回答矛盾制御

エラー条件

優先順位	条件名	条件式	発動条件
1	Q6_回答エラー	(((Q6 on 1~3) >= 2) OR ((Q6 on 4~6) >= 2) OR ((Q6 on 7~9) >= 2) OR ((Q6 on 10~12) >= 2))	成立

改ページ

Q7
必須
この保育施設に野外の遊び場（庭・運動場など）がありますか。

1.ある

2.ない

次へ

改ページ

■お子さまの保育施設における外遊びについてお伺いします。

Q8
必須
保育施設における外遊びの際、施設「外」の緑地（例：近所の公園、川沿い・河川敷、農園、大学キャンパス内の緑地、お寺・神社、森、空き地、他の公共・私有緑地など）をどのくらいの頻度で利用していますか。

1.利用しない

2.月に1回以下

3.月に2～3回程度

4.週に1回程度

5.週に2～3回程度

6.ほぼ毎日

7.わからない

次へ

[選択肢] 番号は回答者へは表示しません

改ページ

Q9
必須
お子さまが保育施設における外遊びで、施設「外」の緑地などを利用している時に、利用に支障をきたすような、管理人、他の利用者、近隣住民等とのトラブルがあったことについて、保育施設から聞いたことありますか。

1.聞いたことがない

2.聞いたことがある　詳細がわかれば、教えてください：**(回答必須)(入力制限なし)(200文字まで)**

次へ

改ページ

Q10
必須

保育施設など集団での緑地利用が多い場合、緑地の整備費（芝生管理など）が高くなることがあります。
もし利用している緑地の整備のために寄付制度が設立されることになった場合、実際に支払うとするならばどの程度の定期的な寄付金が手頃だと思いますか。
「寄付しない」と答えた場合、寄付しない・できない理由を教えてください。

1.（子ども1人／利用1回）10円

2.（子ども1人／利用1回）20円

3.（子ども1人／利用1回）30円

4.（子ども1人／利用1回）50円

5.（子ども1人／利用1回）100円

6.寄付しない　理由：　**(回答必須)(入力制限なし)(200文字まで)**

次へ

改ページ

Q11
必須

子どもたち（5歳以下）にとって、外遊びは以下の点でどのくらい重要だと思いますか。

※この設問は、それぞれ横方向（→）にお答えください。

	1. 全く重要でない	2. あまり重要でない	3. どちらでもない	4. 重要	5. 非常に重要
1. 身体的発達・運動能力					
2. 精神的発達・認知発達					
3. 社会的発達					
4. 幸福・ストレス緩和					
5. 想像力					
6. 自然にふれる					
7. 生き物にふれる					
	1. 全く重要でない	2. あまり重要でない	3. どちらでもない	4. 重要	5. 非常に重要
8. 地域の人々との交流					
9. 自由遊び					
10. 水遊び					
11. 自分を自由に表現できる能力					
12. 命の大事さを知る					
13. 農業体験					

次へ

改ページ

Q12 必須　お子さまが保育施設の園外保育活動で利用する緑地での外遊びについて、以下の点においてどのくらい満足していますか。

※この設問は、それぞれ横方向（→）にお答えください。

	1. 全く満足していない	2. あまり満足していない	3. どちらともいえない	4. 満足している	5. 非常に満足している
1. 身体的発達・運動能力					
2. 精神的発達・認知発達					
3. 社会的発達					
4. 幸福・ストレス緩和					
5. 想像力					
6. 自然にふれる					
7. 生き物にふれる					
	1. 全く満足していない	2. あまり満足していない	3. どちらともいえない	4. 満足している	5. 非常に満足している
8. 地域の人々との交流					
9. 自由遊び					
10. 水遊び					
11. 自分を自由に表現できる能力					
12. 命の大事さを知る					
13. 農業体験					

次へ

改ページ

■お子さまが保育施設に通っていない時の外遊びについてお伺いします。

Q13 必須 保育施設での活動以外（ご自宅や親族・知人宅にいる時など）では、お子さまは外遊びで緑地（例：近所の公園、川沿い・河川敷、農園、大学キャンパス内の緑地、お寺・神社、森、空き地、他の公共・私有緑地など）をどの程度の頻度で利用していますか。

1.利用しない

2.月に1回以下

3.月に2～3回程度

4.週に1回程度

5.週に2～3回程度

6.ほぼ毎日

7.わからない

次へ

[選択肢] 番号は回答者へは表示しません

改ページ

Q14
必須

保育施設での活動以外で、子どもがよく利用する緑地での遊びについて、以下の点においてどのくらい満足していますか。

※この設問は、それぞれ横方向（→）にお答えください。

	1. 全く満足していない	2. あまり満足していない	3. どちらともいえない	4. 満足している	5. 非常に満足している
1. 身体的発達・運動能力					
2. 精神的発達・認知発達					
3. 社会的発達					
4. 幸福・ストレス緩和					
5. 想像力					
6. 自然にふれる					
7. 生き物にふれる					
	1. 全く満足していない	2. あまり満足していない	3. どちらともいえない	4. 満足している	5. 非常に満足している
8. 地域の人々との交流					
9. 自由遊び					
10. 水遊び					
11. 自分を自由に表現できる能力					
12. 命の大事さを知る					
13. 農業体験					

次へ

改ページ

その他設定

回答途中保存期間

1時間(クロスデバイスあり)

アンケートにご回答いただき、ありがとうございました。

【保育施設に関するアンケート】の獲得ポイント

◯◯ポイント

閉じる

あなたの中に未来がある。
一般社団法人 日本マーケティング・リサーチ協会
正会員No. 20168
